# A systematic review of the evidence for deprescribing interventions among older people living with frailty

**DOI:** 10.1186/s12877-021-02208-8

**Published:** 2021-04-17

**Authors:** Kinda Ibrahim, Natalie J. Cox, Jennifer M. Stevenson, Stephen Lim, Simon D. S. Fraser, Helen C. Roberts

**Affiliations:** 1 Academic Geriatric Medicine, Faculty of Medicine, University of Southampton, Southampton, UK; 2 NIHR Applied Research Collaboration Wessex, Southampton, UK; 3 Southampton Biomedical Research Centre, University of Southampton and University Hospital Southampton NHS FT, Southampton, UK; 4 Institute of Pharmaceutical Science, King’s College London, London, UK; 5 Pharmacy Department, Guy’s and St. Thomas’ NHS FT, London, UK; 6 Primary Care, Population Science and Medical Education, Faculty of Medicine, University of Southampton, Southampton, UK

**Keywords:** Frailty, Deprescribing, Medication review, Polypharmacy, Inappropriate medications

## Abstract

**Background:**

Older people living with frailty are often exposed to polypharmacy and potential harm from medications. Targeted deprescribing in this population represents an important component of optimizing medication. This systematic review aims to summarise the current evidence for deprescribing among older people living with frailty.

**Methods:**

The literature was searched using Medline, Embase, CINAHL, PsycInfo, Web of Science, and the Cochrane library up to May 2020. Interventional studies with any design or setting were included if they reported deprescribing interventions among people aged 65+ who live with frailty identified using reliable measures. The primary outcome was safety of deprescribing; whereas secondary outcomes included clinical outcomes, medication-related outcomes, feasibility, acceptability and cost-related outcomes. Narrative synthesis was used to summarise findings and study quality was assessed using Joanna Briggs Institute checklists.

**Results:**

Two thousand three hundred twenty-two articles were identified and six (two randomised controlled trials) were included with 657 participants in total (mean age range 79–87 years). Studies were heterogeneous in their designs, settings and outcomes. Deprescribing interventions were pharmacist-led (*n* = 3) or multidisciplinary team-led (*n* = 3). Frailty was identified using several measures and deprescribing was implemented using either explicit or implicit tools or both. Three studies reported safety outcomes and showed no significant changes in adverse events, hospitalisation or mortality rates. Three studies reported positive impact on clinical outcomes including depression, mental health status, function and frailty; with mixed findings on falls and cognition; and no significant impact on quality of life. All studies described medication-related outcomes and reported a reduction in potentially inappropriate medications and total number of medications per-patient. Feasibility of deprescribing was reported in four studies which showed that 72–91% of recommendations made were implemented. Two studies evaluated and reported the acceptability of their interventions and further two described cost saving.

**Conclusion:**

There is a paucity of research about the impact of deprescribing in older people living with frailty. However, included studies suggest that deprescribing could be safe, feasible, well tolerated and can lead to important benefits. Research should now focus on understanding the impact of deprescribing on frailty status in high risk populations.

**Trial registration:**

The review was registered on the international prospective register of systematic reviews (PROSPERO) ID number: CRD42019153367.

## Background

One-third of people aged over 65 years live with multimorbidity and take five or more regular medicines (polypharmacy), increasing to 50% in over 85 year olds [1, 2]. Polypharmacy in older people is associated with increased risk of serious adverse events, falls, cognitive impairment, functional decline, hospitalisation, length of stay and death, [3–5]. Such harms are amplified in older people living with frailty, a complex geriatric syndrome resulting in decreased physiological reserve [6]. In frail older people the harms might outweigh benefits for some medications e.g. intensive blood glucose control in type 2 diabetes, or the known time to benefit exceeds projected life expectancy e.g. statins [7, 8]. Additionally, the goals of drug treatment in older people living with frailty may change compared with older people in general, shifting the focus from reducing the risk of disease and prolonging life to reducing the burden of treatment and maintaining quality of life [9]. The bi-directional relationship between polypharmacy and frailty has been reported. Drugs and frailty might interact through network of connections, including physiological changes, multiple pathologies and chronic diseases, life expectancy and functional or cognitive status [10–14]. Frailty may influence factors such as drug pharmacokinetics and pharmacodynamics, toxicity, and therapeutic efficacy. In turn, these factors may be involved in the development of frailty [15].

The cure for polypharmacy appears simple and involves deprescribing - the process of tapering /dose reduction, stopping, or switching drugs, with the goal of managing polypharmacy and improving outcomes [16]. There has been considerable research conducted on deprescribing since the term was first used in 2003 [17], and more recently there has been a focus on deprescribing for those living with frailty. Several tools have been developed to assist physicians with deprescribing decisions such as STOPPFrail [18, 19]. However, investigation of the impact of deprescribing on those living with frailty has been limited to date.

Several systematic reviews have synthesised the evidence on outcomes of deprescribing interventions among older people in general [20, 21], or defined by setting including care homes [22, 23], primary care and community [24, 25] and hospitals [26]. These reviews reported that deprescribing is feasible, well tolerated, safe, and generally effective in reducing the number of inappropriate prescriptions. However, these reviews either did not include frail older people or frailty was poorly defined in their included studies, for example based on age or setting such as being in a care home with definition subject to international variability [27]. There is an increasing awareness that identifying frail older people or those at risk of frailty using reliable tools should be part of routine clinical practice, to guide appropriate interventions to improve clinical outcomes [28]. The dynamic nature of frailty highlights a potential for preventive and restorative interventions to maintain the capacity for self-care and to prevent disabilities, falls, functional decline, institutionalisation, hospitalisation and death [29]. For example, it could be crucial to identify older people living with frailty and polypharmacy as priority patients for a medication review and deprescribing intervention, which could potentially reduce medication-related harm and improve patients’ outcomes. Using objective, reliable measures to assess frailty in the context of research studies on deprescribing is also important to assess whether study results can be extrapolated to patients with similar scores, or to measure whether frailty status affects response to deprescribing interventions and vice versa.

Therefore, the aim of this systematic review was to explore the safety and impact of deprescribing among older people living with frailty identified by reliable measures.

## Methods

### Data sources and searches

The search strategy was developed with a senior librarian and used the following databases: Medline, Embase, CINAHL, PsycInfo, Web of science, and the Cochrane library from database conception until January 2020. Keywords such as deprescribing, deprescribe*, polypharmacy, inappropriate prescribing were used (see Appendix). Reference lists of retrieved articles were searched for additional relevant studies. The search was re-run in May 2020 but no further eligible papers were retrieved. The review was carried out using the methods recommended by the Preferred Reporting Items for Systematic reviews and Meta-Analyses (PRISMA) statement [30] and was registered on the international prospective register of systematic reviews (PROSPERO) ID number: CRD42019153367.

### Study inclusion

#### Type of studies

We anticipated a small number of studies to explore deprescribing in frail older people. Only interventional studies with any design, setting or language were included (Table 1).

**Table 1:** PICO statement for study inclusion

Population	Older people (mean age 65+) who live with frailty measured objectively by any reliable tool.
Intervention	Studies at any setting and any language that included deprescribing medication review (including tapering/dose reduction, stopping or switching drugs). Deprescribing as the only intervention or part of medication review where deprescribing accounts for at least 50% of changes.
Comparator	Any, or no, comparator considered
Outcomes	Primary outcome: safety of deprescribing Secondary outcomes: clinical outcomes, medication-related outcomes, feasibility of deprescribing, acceptability and cost-related outcomes.

#### Type of participants

We included interventions that targeted an older population with a median age of 65 years and over, who are identified to be frail using reliable measures including but not limited to Fried Frailty Phenotype, FRAIL scale, PRISMA-7, electronic-Frailty Index, Edmonton Frail Scale Gérontopôle Frailty Screening Tool and Clinical Frailty Scale. To be included, studies had to have at least 50% of their study population identified as frail.

#### Type of interventions

Studies involving deprescribing as the only intervention or as part of medication review intervention where deprescribing accounts for at least 50% of the total recommendations were included. Studies where deprescribing formed part of a multi-dimensional intervention (such as in combination with nutritional and physical activity components) were excluded as it is difficult to ascertain which component of the intervention was responsible for the reported outcomes.

#### Type of outcomes

The primary outcome we chose was safety of deprescribing. We defined safety in terms of reported adverse events, hospital admission and/or all-cause mortality.

Secondary outcomes included clinical outcomes (such as frailty status, function, falls, cognition, depression, quality of life), medication-related outcomes (such as changes in number of medications and Potentially Inappropriate Medications PIMs), feasibility of deprescribing (defined by the number of patients/proportion who successfully stopped medications), its acceptability by patients or healthcare practitioners, and cost-related outcomes.

### Study selection

Two authors (KI & NC) independently screened the title and abstracts of identified articles using the Rayyan electronic platform to identify studies that met the inclusion criteria [31]. Following each stage, any disagreement was resolved by discussion.

### Quality assessment

Study quality was assessed separately by two authors (SL & SF) using the standardized Joanna Briggs Institute checklists for each study type, with total scores of 13 for randomized controlled trials (RCTs) and 9 for non-randomised experimental studies. Final scoring was agreed by discussion. A score ≥ 7/13 for RCTs and 5/9 for non-randomised experimental trials were considered to represent good quality.

### Data abstraction and synthesis

Due to heterogeneity of study designs and outcome measures, quantitative synthesis (meta-analysis) was not possible and narrative synthesis of the findings was conducted following the Synthesis Without Meta-analysis (SWiM) guideline [32]. Data from included studies were extracted independently by two authors (KI & JS) into a pre-defined template for conceptualisation and construction of the literature review (Table 2). Data abstracted included: year of publication, country, setting, number and age of participants, description of the deprescribing intervention and any comparator, types and classes of medications most frequently deprescribed, frailty measures used, deprescribing tools and outcomes of deprescribing. Studies were grouped according to intervention type (pharmacist-led or multidisciplinary team-led) due to the heterogeneity of study designs and outcomes. Outcome data were summarised for each study and compared.

**Table 2:** Study characteristics

Intervention	Authors and year of publication	Study design	Setting and country	N of Participants (age and gender)	Description of the intervention	medication commonly stopped or reduced	Comparator (if any)	Study Follow up	Frailty tool	Deprescribing tool	Outcome measures	Quality scoring
Pharmacists-led medication review	Khera S 2019 [33]	Quasi-experiment (pre- post- comparison)	Primary care Canada	*N* = 54 Age: 81 ± 6.74 Gender: F 61%	Structured medication review in a primary care team-based seniors’ program. Medication review that included (1) preparing draft medication plan;(2) meet with patient and caregiver; (3) finalise medication plan/report; (4) following up	NR	Pre- and post- intervention	12 months	Electronic Frailty Index (e-FI)	STOPP/START and Beers criteria in addition to pharmacist’s expertise	**Primary outcomes**: NA **Secondary outcomes** *Medication-related outcomes:* No significant changes in total number (12.1 meds pre to 11.7meds post, *P* = .254). Significant decrease in number of PIMs (1.15 meds pre to 0.9 meds post; *P* = .006). A statistically significant correlation between number of PIMs and frailty (r = 0.280, *P* = .040). *Feasibility* 72% of the deprescribing recommendations were implemented	7/9
	Ailabouni N 2019 [34]	Quasi-experiment (pre- and post- comparison)	Three Residential Care Facilities New Zealand	*N* = 46 Age: not specified (inclusion ≥65 years old) Gender: F 74% (*n* = 34)	Patient-centred collaborative pharmacist-led medication review with GPs including (Medical history, initial consultation, deprescribing medication review, development of medication management plan, monitoring and follow up	anticholinergic and sedative medicines (tramadol, codeine, citalopram, Escitalopram, amitriptyline)	Same participants before the intervention (pre-post design)	6 months	the Edmonton Frailty Scale	peer-reviewed deprescribing guidelines of sedatives and anticholinergic medicines developed by the research team	**Primary outcomes:** Significant decrease in -adverse drug reactions by a mean difference of 2.8 (95% CI; *p* < 0.05) and 4.2 (95% CI; *p* < 0.05) 3 and 6 months after deprescribing -Psychiatric adverse effects decreased by a mean difference of 1.8 (*p* < 0.05; 95%, CI) and 2.24 (*p* < 0.05; 95%, CI) after 3 and 6 months **Secondary outcomes** *Clinical outcomes:* No significant changes in cognition or QoL 6 months after deprescribing. Significant decrease in -number of falls 6 months post deprescribing. -depression scores (Median difference: − 2; *p* < 0.05). -frailty scores 6 months after deprescribing (mean difference = 1.35 (*p* < 0.05,95%, CI). *Medication-related outcomes*: Significant reduction in: -total N of meds (2.13 medicines per patient) -Drug burden index scores by 0.34 6 months after deprescribing Feasibility 72% of the recommendations agreed and implemented	6/9
	Whitty R 2018 [35]	Prospective interventional cohort study	Hospital (General internal medicine ward) Canada	104 (Intervention = 53 Control = 51 Age: intervention 79.6 ± 11.7 years control 79.2 ± 13.4 years Gender: intervention F 43% Control F 63%	1.Medication review by the Medication Rationalization team MERA (pharmacists and physicians) using guideline-based algorithm. 2.Their recommendations to stop, or change dose of medicines then reviewed by the ward pharmacist and physician.	vitamins/minerals, lipid-lowering agents, herbal supplements, proton pump inhibitors, docusate, Antiplatelets Benzodiazepines, Bisphosphonates, Dihydropyridine, Opioids	eligible patients admitted concurrently to general internal medicine wards where the intervention was not delivered	Follow up: 3 months after discharge	Clinical Frailty Score Score ≥ 4	a guideline- based algorithm based on STOPP guidelines, Beers criteria, Choosing Wisely, and choosing wisely Canada	**Primary Outcomes**: NA **Secondary outcomes** *Medication-related outcomes:* Reduction in number of medications by a mean of 3 per patient, Significant decrease in number PIMs (3.1 intervention v. 0.9 control meds per patient, *p* < 0.01). *Feasibility* 81% of total recommendations were accepted by the admitting physician *Acceptability* 87% participants felt comfortable stopping medications as recommended by the team and only a very small number found the experience stressful or confusing (5 and 11% respectively) *Cost-related outcomes*: The total direct cost saved of stopped medications was $1508.47, or $94.28 per 100 patient-days.	6/9
Multidisciplinary team (MDT)-led medication review intervention	Curtin D 2019 [36]	Randomized controlled trial	Hospital (in transition to care homes) Ireland	*N* = 130 (65 in the intervention group, 65 in the control group) Age = 85.1 (±5.7) Gender = 61% F.	Single pre-discharge STOPPFrail-guided deprescribing. The research Physician recommended a medication withdrawal plan to one of the participant’s attending physicians and also documented in the patient’s medical record. The attending physician judged whether or not to accept the drug withdrawal plan and implement the recommended changes.	Lipid lowering therapies, neuroleptic antipsychotics, proton pump inhibitors, antiresorptive/bone anabolic drugs, calcium supplementation, and multivitamin combination supplements.	Usual pharmaceutical care	3 months	Clinical Frailty Index (CFS score ≥ 7)	STOPPFrail	**Primary outcomes:** No significant difference between groups for hospitalisation, mortality **Secondary outcomes:** *Clinical outcomes:* No significant differences between the groups in incident of falls or fractures. QoL deteriorated significantly in both the intervention and control groups from baseline to 3-months, but no statistically significant differences were found between the groups *Medication-related outcomes* Reduction in the number of meds of 2.6 in the intervention group vs 0.36 in the control group (*p* < 0.001). *Cost-related outcomes* mean difference in the monthly medications cost of $61.74 ± $26.60; 95% CI; *P* = 0.02) between the intervention and the control groups.	10/13
	Garfinkel D 2017 [37]	A longitudinal, prospective, nonrandomized study	Community dwelling people Israel	177 (122 in the intervention group and 55 in the comparator group) 64% female) Age: 83.4 ± 5.3 in the PDP group, and 80.8 ± 6.3 in the NR group Gender: F 64%	Medication review performed at home by a geriatrician to implement poly-deprescribing (PDP) of 3 or more drugs in collaboration with GPs. it combines ethics, EBM and clinical judgement while giving the highest priority to patient/ family preferences.	Statins, aspirin, benzodiazepines, proton pump inhibitor, antihypertension drugs, SSRI/SNRIs	The non-response group (NR) including participants who agreed to stop only 2 medication or less	follow up: 3 years	Fried frailty phenotype	concurrent de-prescribing of multiple medications based on the Garfinkel algorithm	**Primary Outcomes:** The number of major complications was significantly reduced (*p* < 0.002 in all). The rate of hospitalizations (RR = 0.86, 95% CI: 0.58–1.29) and survival (66.7%} 6.4% for the control group versus 77%} 3.8% for the intervention group) were comparable **Secondary outcomes** *Clinical outcomes* The PDP group showed significantly less deterioration (sometimes improvement) in health parameter: functional (OR = 4.63, 95% CI: 2–10.72), mental (OR = 60.41,95% CI: 16.38–222.82) and cognitive status (OR = 4.394, 95% CI: 1.93–9.98) night sleep quality (OR = 10.65, 95% CI: 4.06–27.96), daily alertness (OR = 5.38, 95% CI: 1.95–14.84), appetite (OR = 15.89, 95% CI: 4.91–51.45), and sphincter control (OR = 6.77, 95% CI: 2.54–18.03). *Medication-related outcomes*: the median number of drugs reduced from 11 to 4 in PDP group (*p* <0.0001). *Feasibility* 91% of the recommendations made by a geriatrician were accepted by GPs *Acceptability* The overall satisfaction of patient/family from the changes was defined as high/very high in 89% (*n* = 109)	6/9
	O. Dalleur 2014 [38]	Randomised controlled trial	Hospital, Belgium	146 (intervention = 74, control group = 72) Median age = 85 (IQR 81–88) Gender: F 63%	Medication review on admission using STOPP criteria by inpatient geriatric consultation team (consisting of nurses, geriatricians, a dietician, an occupational therapist, a physiotherapist, a speech therapist, and a psychologist) who makes recommendation to the hospital physician for the discontinuation of PIMs.	Benzodiazepines, antiplatelet, b-blockers, tricyclic antidepressants, and neuroleptics	Non-matched randomised control group (usual care without using STOPP tool)	Follow up: 12 months (only for those with PIMs identified, 50 patients; 26 in intervention group and 24 in the control group)	Identification of Seniors At Risk (ISAR) score of ≥2/6 Median score = 3	STOPP criteria	**Primary outcomes:** NA **Secondary outcomes** *Medication-related outcomes* At discharge, the reduction in PIMs was twice as high for the intervention group as for the control group (39.7 and 19.3%, respectively; [OR] 2.75 [95% CI = 1.22–6.24]; *p* = 0.013). PIM discontinuation rate of benzodiazepines tended to be higher in the intervention than in the control group (34.6 vs. 6.7%; *p* = 0.063) At the patient level, the reduction in the prevalence of PIMs (i.e. patients having one or more PIM) did not differ between the intervention group and the control group (23.1 vs. 16.1%; OR 1.5 [95% CI 0.49–4.89]; *p* = 0.454). However, the proportion of patients with at least one improvement to their drug treatment was higher for the intervention group than for the control group (25.7 vs. 13.9%; *p* = 0.034).	10/13

*N* Number, *SD* Standard Deviation, *F* Female, *NR* Not Relevant, *PIMs* Potentially Inappropriate Medications, *GP* General Practitioner, *QoL* Quality of Life

## Results

**Fig. 1:**
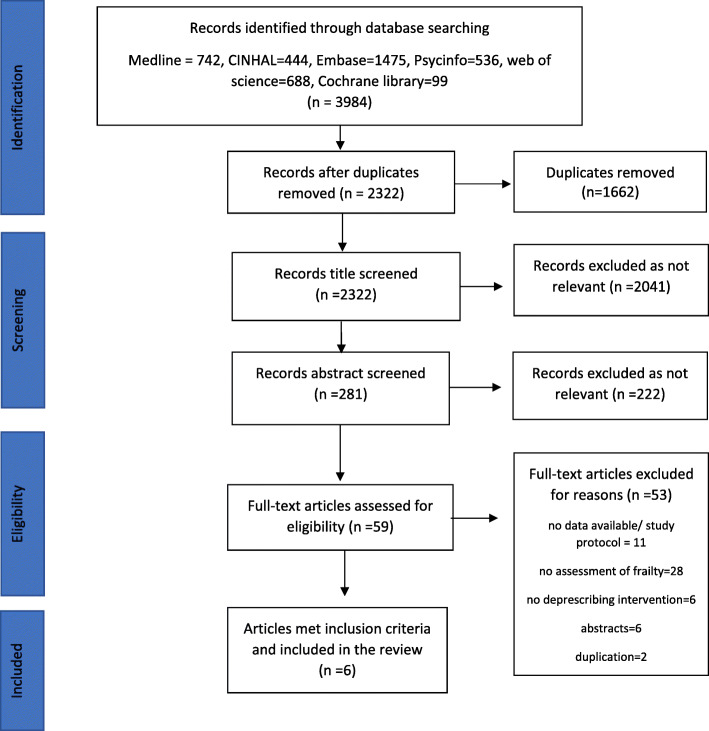
PRISMA Flow Diagram of identification of articles

Two thousand three hundred twenty-two articles were identified, and 57 articles were selected for full text assessment from which six journal articles were included in this review (Fig. 1). Six conference abstracts were excluded due to limited information available and poor quality of reporting. No articles in any language other than English were identified. The quality of the full text articles included was good: ≥6/9 for the four non-randomised experimental studies and 10/13 for the two randomised-controlled trials RCTs.

### Participants characteristics

The total number of participants in all included studies was 657, while individual sample sizes ranging from 46 to 177 and mean participant age range from 79 to 85 years. The Canadian Clinical Frailty Score (CFS) was the most commonly used frailty measure (*n* = 2) [35, 36], with others including: the Edmonton Frailty Scale [34], Identification of Seniors At Risk (ISAR) [38], the Electronic Frailty Index (e-FI) [33], and Fried Frailty Phenotype [37].

### Study characteristics and deprescribing interventions

This review includes two RCTs, two pre- and post- comparison studies and two prospective interventional cohort studies (Table 2). Studies were conducted in Ireland, Belgium, New Zealand, Canada, and Israel. Study settings included: hospital (3), care home (1), primary care (1), community (1). All included articles were published between 2014 and 2019.

Several tools and algorithms were used to guide deprescribing. Two studies used explicit criteria (lists of drug names targeted); one used the STOPP criteria [38], and one used the STOPPFrail tool [36]. Two studies utilised implicit criteria (lists of evaluative questions or a process) developed by the research teams themselves including the Garfinkel algorithm for concurrent deprescribing of multiple medications [37], and guidelines for targeted deprescribing of anticholinergic and sedative medicines [34]. The remaining two studies used a combination of both explicit and implicit criteria; one used STOPP and Beer’s alongside pharmacist’s judgment [33], and the second used an algorithm based on STOPP guidelines, Beers criteria, Choose Wisely and Choose Wisely Canada [35]. The medications most frequently deprescribed across the studies regardless of the setting were: benzodiazepines, antidepressants, neuroleptics, opiates, lipid-lowering agents (statins), vitamin and nutritional supplements, proton pump inhibitors, and cardiovascular drugs (aspirin, antiplatelets, b-blockers, digoxin).

Due to heterogeneity of the outcome measures and study designs, studies were grouped according to interventions: pharmacist-led deprescribing interventions (*n* = 3) and multidisciplinary team-led intervention (*n* = 3).

#### Pharmacist-led deprescribing

Three of the six studies described pharmacist-led deprescribing interventions: one across three care home facilities [34], one in primary care (across six practices) [33] and one in hospital [35]. The three studies were non-randomised experimental studies with a good quality score, > 6/9. Follow up periods were 3 months (after discharge from hospital), 6 months (in primary care) and twelve months (in care home).

The care home and primary care studies used a person-centred, collaborative, pharmacist-led deprescribing medication review with the General Practitioner (GP) [33, 34]. Both interventions involved a detailed medication review process that engaged patients and their relatives in decisions about medication discontinuation; both drafted and shared a medication plan and followed patients for close monitoring. While the hospital study was a prospective interventional cohort study that employed medication reviews by a Medication Rationalization (MERA) team, involving physicians and led by pharmacists, for 53 frail inpatients (CFS ≥4). These were compared with 51 patients in the control arm who did not receive the MERA review [35].

In the care home study, the pharmacist used implicit guidelines of sedatives and anticholinergic medicines developed by the research team among 46 residents identified to be frail using Edmonton Frailty Scale [34]. Whereas in the two studies in primary and secondary care, pharmacists used the STOPP and Beers criteria to identify prescribing problems among 54 community dwelling older people with some degree of frailty using the electronic frailty index (e-FI) [33] and 53 frail inpatients using the Clinical Frailty Score (CFS ≥4) [35].

#### Multidisciplinary team (MDT)-led deprescribing

Three studies implemented multidisciplinary team-led deprescribing focused medication review: two were RCTs in hospital settings (good quality score of 10/13) and one was a longitudinal prospective interventional study in community (good quality, 6/9). Follow up periods were three and twelve months in hospital studies and 3 years in the community study.

One RCT of 146 patients (74 in the intervention group vs 72 in the control group) living with frailty (ISAR ≥2/6) implemented medication review using STOPP criteria on admission by an inpatient geriatric team consisting of nurses, geriatricians, a dietician, an occupational therapist, a physiotherapist, a speech therapist, and a psychologist [38]. Another RCT of 130 inpatients (65 in the intervention group vs 65 in the control group) living with frailty (CFS score ≥ 7) used a STOPPFrail-guided deprescribing intervention by a research physician pre-discharge to a care home [36]. A longitudinal prospective nonrandomized study among 177 community dwelling older people living with frailty (median Fried Frailty Phenotype = 3) employed concurrent deprescribing of multiple medications based on the Garfinkel algorithm by a geriatric consultant in collaboration with GPs [37]. The intervention group included patients who had 3 or more medications deprescribed (Poly-De-Prescribing PDP group *n* = 122), while the control group included participants who agreed to stop only 2 medications or less (*n* = 55).

### Outcomes of deprescribing

The outcomes reported in the included studies are presented in Table 2; safety of deprescribing in three studies; clinical outcomes (*n* = 3); medication-related outcomes (*n* = 6); feasibility of deprescribing (*n* = 4); acceptability (*n* = 2) and cost-related outcomes (*n* = 2).

#### Primary outcome

##### Safety of deprescribing

Three studies reported the safety of deprescribing and its impact on adverse events [34, 36, 37]. Potential adverse drug reactions using the UKU-SERS score, in a pharmacist-led deprescribing intervention among 46 care home residents, decreased by a mean difference of 2.8 (95% CI; *p* < 0.05) after 3 months and 4.2 (95% CI; *p* < 0.05) after 6 months of deprescribing of sedative and antipsychotic medications [34]. In addition, adverse effects of psychotropic medications decreased significantly by a mean difference of 1.8 (95%, CI; *p* < 0.05) 3 months after deprescribing, and by a mean difference of 2.24 (95%, CI; *p* < 0.05) after 6 months of deprescribing. One RCT in hospital reported that 88% of deprescribing recommendations based on STOPPFrail were accepted and implemented and no adverse events were reported of MDT-led deprescribing during 3 months follow-up [36].

Two MDT-led deprescribing studies in hospital and community showed no significant differences in unplanned hospitalisation and mortality [36, 37]. The RCT in hospital showed no statistically significant differences between the intervention and control groups for 3 months unscheduled hospital presentations (0.14 intervention vs 0.08 control, 95% CI, *P* = 0.27) and mortality (0.18 vs 0.28, 95% CI; *P* = 0.22) [36] . Similarly a longitudinal cohort study in community reported that the incidence of hospitalisations per patient per year (0.39 intervention vs 1.02 comparator, *p* = 0.1006) and survival (77% intervention vs 67% comparator group, *p* = 0.026) was comparable between the groups after 3 years [37]. This study also reported that the incidence of significant complications per patient/year was significantly reduced in the PDP group [0.22 intervention vs 1.72 comparator group, *p* = 0.0047] [37].

#### Secondary outcomes

##### Clinical outcomes

*Frailty and function:* Two studies reported the outcomes of deprescribing on frailty and function [34, 37]. Pharmacist-led deprescribing sedatives and anticholinergic medicines among 46 care home residents showed a significant decrease in frailty scoring (mean difference of 1.35, 95% CI, *P* < 0.05) using the Edmonton Frailty scale, after 6 months of deprescribing. Another pharmacist-led deprescribing study did not report whether deprescribing led to changes in frailty status but reported a positive and statistically significant correlation between number of PIMs (using STOPP and Beers criteria) and frailty using e-FI (r = 0.280, *P* = .040) [33]. The impact of deprescribing on functional status defined using a 5 point scale (1 = independent, 2 = frail, 3 = mild disability, 4 = disability, 5 = severe disability) was examined in another MDT-led deprescribing study [37]. Patients in the poly-deprescribing group had less functional deterioration compared to the comparator group [37] (69.1%) vs 42(34.4%), *P* < 0.001).

*Falls:* The impact on falls was mixed on reports from two studies [34, 36]. Significant decreases in falls rate, defined as the number of falls in the past 90 days, was reported after pharmacist-led deprescribing psychotropic medicines among care home residents [34]. But on the other hand, falls risk (determined using an in-house falls risk assessment tool utilised by most residential care facilities in New Zealand) remained the same 6 months after deprescribing in the same study. Another MDT-led deprescribing RCT study using STOPPFrail at hospital discharge reported no significant difference in incidence of falls (0.27 vs 0.30, 95% CI, *P* = 0.75) and non-vertebral fractures (0.02 vs 0.09, 95%, *P* = 0.18), among patients who moved to nursing home after 3 months of deprescribing [36].

*Cognition, depression and mental health status:* two studies reported outcomes on cognition, depression and mental status [34, 37]. No change in cognition using the interRAI cognitive performance scale was observed after 3 and 6 months of pharmacist-led deprescribing of antipsychotic medications among care home residents (mean difference of 0, *p* = 0.26). However, significant improvement of depression scores using the geriatric depression scale (GDS) (mean difference of − 2, *p* < 0.05) were seen after 6 months [34]. Another MDT-led deprescribing study reported that patients in the poly-deprescribing (PDP) group had improvement in mental status using the Mini Mental State Examination (MMSE) test (3 comparator vs 63 intervention, *p* < 0.0001) and cognitive status (0 comparator vs 7 intervention, *P* = 0.0004) [37]. These improvements occurred within 3 months after deprescribing in 83% and persisted for ⩾2 years in 68%.

*Quality of life (QoL):* Two studies assessed QoL of participants and showed no significant differences after implementing deprescribing interventions [34, 36]. QoL among care home residents was assessed using EQ-5D-3L and showed no significant difference pre and 6 months post deprescribing [34]. QoL in one RCT in hospital using QUALIDEM or ICECAP-O scores showed deterioration in both the intervention and the control group from baseline to 3 months follow-up, but no statistically significant differences were found in the mean changes between groups [36].

##### Medication-related outcomes

All six studies reported medication-related outcomes [33–38]. Four studies reported a significant reduction in the number of medications taken by patients living with frailty after implementing deprescribing, ranging from a mean of 2–3 medicines stopped per patient [34–36] across the different settings to unsurprisingly 7 medications per patient when poly-deprescribing of three or more drugs was implemented in people’s home [37]. Two studies also reported significant decreases of potentially inappropriate medications associated with deprescribing interventions [33, 38]; for example the deprescribing interventions in two hospital studies reported a mean decrease in number of PIMs of 2.2 (*p* < 0.01) in a pharmacist-led study and reduction in PIMs was twice as high for the intervention group compared to the control group in a MDT-led deprescribing intervention. One care home study reported a significant decrease in the drug burden index (by 0.34) 6 months after deprescribing in care home residents with a pharmacist-led deprescribing intervention [34].

##### Feasibility of deprescribing

Four studies reported the feasibility of deprescribing among older people with frailty [34–37]. They displayed that 72–91% of the suggestions to deprescribe medications made by either pharmacists or the MDT were accepted and implemented across the different settings [34–37]. For example, in care homes, forty-five PIMs were identified and suggested to be stopped by pharmacists, of which 82% were agreed upon by the residents’ GP and 96% were agreed upon by the resident or their relatives/family resulting in the implementation of 72% of the recommendations [34]. Similarly in two hospital studies, 72 and 81% of the recommendations made were accepted and implemented by the admitting physician and then patients [35, 36]. In one community study, 91% of the recommendations made by a geriatrician were accepted by GPs [37].

Deprescribing was also reported to be well tolerated as most medications stopped were not restarted. For example, in care homes, medicines were re-prescribed by the GP in only five instances (15%); stopping medication was not completed in 13 residents (28%) due to mood changes, increased pain levels or overall health deterioration [34]. Similarly in hospital, of the 162 medications that were stopped only 40 (25%) were restarted during hospital admission or at time of discharge and 81% of medications stopped during hospitalisation remained discontinued after 3 months [35]. Another RCT study among hospitalised older patients discharged to care homes showed that only three medications stopped at discharge by the MDT were restarted [36].

##### Acceptability of deprescribing

Two studies evaluated the acceptability of their deprescribing interventions and showed that patients and healthcare professionals were happy to stop unnecessary medication [35, 37]. For example, following a pharmacist-led intervention 87% participants felt comfortable stopping medications as recommended by the team and only a small number found the experience stressful or confusing (5 and 11% respectively) [35]. In the poly-deprescribing intervention in community, the overall satisfaction of patient/family from the changes was defined as high/very high in 89% [37].

##### Cost-related outcomes

Two studies reported the cost implications of deprescribing [35, 36]. A pharmacist-led intervention reported a total saving of $1508.47 or $94.28 per 100 patient-days when STOPP criteria were implemented in hospital [35]. Use of STOPPFrail by an MDT at discharge from hospital also led to a mean change in monthly medication cost of –$74.97 compared to –$13.22 in the control group (mean difference $61.74; 95% CI; *P* = .02) [36].

## Discussion

This review expands on prior literature reviews by synthesising studies on medication deprescribing that specifically addressed older people living with frailty, as they are more vulnerable to the adverse effects of medicines compared to older people in general. Only six studies (two were RCTs) with overall good quality that reported the outcomes of deprescribing interventions among older people, with reliably identified frailty, were found. The outcomes of deprescribing in older people living with frailty were similar to those reported in older people in general in terms of feasibility, acceptability and safety, as mortality and hospitalisation rates did not increase after stopping medications. Deprescribing interventions led to a significant reduction in the number of medications and PIMs with potential cost saving. Included studies also suggest some evidence of potential improvements in function, frailty status, mental health and depression scores. Outcomes did not differ when the intervention was led by a pharmacist or MDT including mainly medical practitioners and whether explicit or implicit criteria were used. But the heterogeneous study designs limit our ability to make firm conclusions regarding this matter.

Deprescribing medications has raised some ethical dilemmas and fear of negative outcomes has been reported by prescribers as a barrier to deprescribing [39]. Among older people with identified frailty, there is some evidence from the included studies in this review that deprescribing is safe, as it did not adversely change hospitalisation and mortality rates. A number of systematic reviews have investigated the impact of deprescribing on mortality among general population of older people; one reported that deprescribing reduced mortality in non-randomized studies but no changes were observed in RCTs [40]; other reviews suggested a reduction in all-cause mortality with deprescribing interventions in nursing home residents [22, 23]. We reported some evidence that deprescribing is feasible and well tolerated by older people living with frailty and is acceptable by healthcare professionals and patients, which is in agreement with existing studies in older people in general [41, 42]. In our review we identified that 72–91% of recommendations made were implemented and very few patients (25%) restarted their medications. A recent review of 26 papers reported the proportion of patients who successfully stopped their medication varied from 20 to 100% and in 19 studies the proportion was > 50% [24]. The feasibility and safety of deprescribing should encourage clinicians to regularly discuss the decision to continue or deprescribe chronic medications with their patients living with frailty, following a patient-centred, structured deprescribing process with planning, tapering and close monitoring during, and after medication withdrawal.

Few studies in the review reported clinical outcomes such as frailty, falls, cognition and depression; with more focus placed on the success of the interventions in reducing number of medications and especially inappropriate ones. This focus on process and lack of clinical outcome data with inconsistency in outcome measurement have also been highlighted as limitations in deprescribing studies to date. A 2017 review of deprescribing interventional studies among older people in general reported the outcome measures most commonly used were number of medications or PIMs stopped, healthcare use, and adverse events [43]. Patient-reported outcomes, geriatric syndromes (e.g. falls, fractures, gait speed, depression and delirium) or cost evaluations were infrequently reported, and frailty was not used as either inclusion criteria or an outcome measure. There is no consensus among researchers and clinicians on appropriate outcomes of deprescribing and more research is needed in this area. Frailty should be considered as an outcome in deprescribing interventions in older people and the focus should be placed on understanding the impact of deprescribing on frailty trajectory.

The strong relationship between polypharmacy and frailty and the potential to reverse frailty status [44, 45], makes it important to understand the impact of deprescribing on frailty. Only one study included in our review examined the impact of deprescribing on frailty status among 46 care home residents using the Edmonton frailty tool and reported positive results [34]. The Edmonton frailty tool consists of 9 domains including number of medications [46]. It is unclear from the study which domains were influenced by the deprescribing intervention or to what extent the improvement could simply reflect a decrease in the number of medications used. Another included study reported that frailty and PIMs were significantly correlated but did not report the impact of deprescribing on frailty status. There is a lack of research on the impact of stopping medications on frailty status but some current registered clinical trials propose to measure this relationship [47, 48]. It is also important to understand the mechanism by which deprescribing might influence frailty via functional or cognitive changes or through other possible mechanisms.

No effect of deprescribing on the quality of life among older people with frailty was reported in our review. These findings are consistent with literature published in older people in general [20, 21, 49, 50]. Possible explanations for this might be that the impact of deprescribing on QoL may depend on the specific combination of medication(s), patient population and patients’ preferences, clinical setting, timing of QoL measurement or the QoL measurement tools used. We found a positive impact of deprescribing sedative and psychotic medications using a specific algorithm on rate of falls among older care home residents living with frailty, but no similar impact was obtained when the STOPPFrail tool was used among hospitalised patients discharged to care homes. This might be explained by the fact that the deprescribing algorithm focused on sedative and psychotic medications resulting in a higher proportion of anticholinergic medications being stopped compared to a tool with a broader remit like STOPPFrail. The inconsistency in reported findings regarding the relationship between falls and deprescribing is clear in the literature. For example, a recent review published in 2017 reported that falls-risk drug withdrawal strategies did not significantly change the rate of falls, number of people who fell or rate of fall-related injuries over a 6 to 12 months follow-up period in five included papers [51]. However, another review suggested that deprescribing interventions could significantly reduce the number of people who fall in care homes by 24% [22]. They related this to the significant reduction in number of residents on PIMs by 60% such as anticholinergics, which have been consistently associated with cognitive impairment and falling in older people. As we mentioned above, the impact of deprescribing on falls could be mediated by the deprescribing tools used and further research should explore this relationship.

The intervention process, who led deprescribing or the deprescribing tools used, appeared to have no differing effects in reducing unnecessary medications in our review. But the heterogeneity in study designs and the small number of included studies limit our ability to conclude whether one approach is more or less effective than another. Other reviews suggested that pharmacist-led deprescribing intervention in older people in general were more effective in reducing unnecessary medications compared to interdisciplinary team interventions [52, 53]. The concurrent use of both explicit lists of potentially inappropriate medications and systematic appraisal of every medication taken was suggested to help improve complex regimes [54]. Deprescribing techniques may be guided by the clinical situation. Stopping medicines one at a time might be most appropriate for managing people whose health status is stable in out-patient settings, whereas ‘concurrent deprescribing’ of multiple medications may be more appropriate for inpatients where it is easier to monitor for withdrawal effects [54]. It is also recommended to use deprescribing as a ‘drug holiday trial’ as sometimes drugs will need to be restarted when symptoms recur or withdrawal effects are experienced, which necessitates monitoring and follow up [54].

This review is the first to summarise the evidence and impact of deprescribing among older people identified as living with frailty. Most published reviews focused on the general population of older people or in a specific setting. With the increasing awareness of the importance of identifying frailty using reliable measures to allow implementation of effective interventions, this review expands our knowledge of the evidence of deprescribing among this population who are more vulnerable to harm from medications. However, there were several limitations in our review. The inclusion criteria required a reliable and valid measure of frailty. This is important to allow extrapolation of the study results to patients with similar scores, or to measure whether frailty status affects response to deprescribing interventions. However, we may have excluded articles assessing frail older people but which utilised less specific methods of assessing frailty or those that assumed frailty depending on age or setting such as studies in care homes. Multicomponent interventions including deprescribing or medication review where deprescribing accounted for less than half of the recommendations were excluded, as our aim was to understand the evidence and impact of deprescribing among those living with frailty. We did not search the grey literature and may have missed some additional resources. Although we followed SWiM criteria, our synthesis of the studies should be treated with caution because of the limited number of included studies and their heterogeneity. We were also unable to perform a meta-analysis because of the heterogeneity of outcomes within the included studies.

## Conclusion

This review highlights the paucity of published literature on deprescribing among older people living with frailty. The included studies used objective frailty measures and thus may not capture all studies that included frail older people. Studies were heterogenous in their settings, designs and outcomes reported making it difficult to make definite conclusions. However, we suggest that deprescribing could be safe, feasible, well tolerated and can lead to important benefits on geriatric conditions such as depression, function and frailty. Deprescribing interventions in this review appear to be effective whether led by pharmacists or multidisciplinary teams using explicit or implicit tools. This has implications for clinical practice as deprescribing could be effectively led by pharmacists in liaison with GPs in community settings, whereas multidisciplinary teams (with or without access to pharmacists) could play a key role in deprescribing in acute settings. However, more research is needed in the area of deprescribing and frailty and future studies should include those living with frailty in their samples. Moreover, in order to address the gap in our understanding of the effectiveness of deprescribing interventions on reducing and reversing frailty, or stopping its progression, adequately powered randomised controlled trials that include reliable measures of frailty should be conducted.

## Data Availability

The datasets used and/or analysed during the current study are available from the corresponding author on reasonable request.

## Appendix

### Search strategy

1. exp. aged/
2. (elder* or geriatric* or older* or aged or aging or ageing).mp. [mp = title, abstract, heading word, drug trade name, original title, device manufacturer, drug manufacturer, device trade name, keyword, floating subheading word, candidate term word]
3. 1 or 2
4. frailty/
5. frail*.mp. [mp = title, abstract, heading word, drug trade name, original title, device manufacturer, drug manufacturer, device trade name, keyword, floating subheading word, candidate term word]
6. 4 or 5
7. 3 and 6
8. deprescription/
9. inappropriate prescribing/
10. polypharmacy/
11. (deprescrib* or deprescription* or polypharmacy or de-prescrib* or de-prescription* or poly-pharmacy).mp. [mp = title, abstract, heading word, drug trade name, original title, device manufacturer, drug manufacturer, device trade name, keyword, floating subheading word, candidate term word]
12. 8 or 9 or 10 or 11
13. 7 and 12

## Abbreviations

PRISMA: The Preferred Reporting Items for Systematic reviews and Meta-Analyses
PROSPERO: The international prospective register of systematic reviews
PIMs: Potentially Inappropriate Medications
RCTs: Randomized controlled trials
SWiM: The Synthesis Without Meta-analysis
CFS: The Canadian Clinical Frailty Score
ISAR: Identification of Seniors At Risk
e-FI: The Electronic Frailty Index
GP: General Practitioner
MERA: Medication Rationalization
MDT: Multidisciplinary team
PDP: Poly-De-Prescribing
GDS: The geriatric depression scale
MMSE: Mini Mental State Examination
QoL: Quality of life
